# Reivindicando poder dibujar el árbol familiar en la historia clínica electrónica de atención primaria

**DOI:** 10.1016/j.aprim.2023.102768

**Published:** 2023-09-20

**Authors:** Ismael Ejarque Doménech, Carolina Cuenca Valero, Itziar Reinoso Fernández, Ana María García Rodríguez

**Affiliations:** a Centro de Salud de Almácera, Valencia, España; b Consulta de Genética Clínica, Hospital Vithas Aguas Vivas de Alzira, Valencia, España; c Centro de Salud de Tres Forques, Valencia, España; d Servicio de Urgencias, Hospital de Requena, Valencia, España; e Centro de Salud Sama de Langreo, Asturias, España; f Hospital Valle del Nalón, Asturias, España; g Centro de Salud Delicias I, Valladolid, España; h Facultad de Medicina, Valladolid, España

*Sr. Editor,*

Las enfermedades raras son de causa genética en aproximadamente 80% de los casos. Por tanto, es crucial que el médico especialista en Medicina Familiar y Comunitaria conozca este hecho y se forme adecuadamente en genética clínica para atender con excelencia a estos pacientes, pues los médicos de familia somos la puerta de entrada al Sistema Nacional de Salud y el profesional que mejor conoce la historia personal y familiar del paciente.

En el último borrador del Programa Formativo de Medicina Familiar y Comunitaria de 2023 ya se contemplan las habilidades y competencias en genética clínica para los futuros residentes de la especialidad. Esto supone que cuando finalicen su formación deben tener la destreza suficiente para enfrentarse al manejo de pacientes afectos por una enfermedad rara. Además, sus tutores y el resto de los médicos adjuntos de la especialidad tendrán que actualizarse en ello.

Un aspecto muy importante que reivindicamos aquí y ahora es el hecho de que en la historia clínica electrónica de atención primaria de la inmensa mayoría de las 17 + 2 Comunidades Autónomas españolas no se contempla la posibilidad de poder llevar a cabo la ilustración del árbol familiar de los pacientes de su cupo ni de su genograma^1^. El genograma ofrece información sobre relaciones sociales y el árbol familiar acerca de relaciones biológicas (genéticas) y ambos deberían estar disponibles, de ahí la importancia de dotar de un soporte informático adecuado de gráficos con una información tan relevante en la historia clínica del paciente y de su familia. También sería deseable si se pudieran aplicar técnicas de Inteligencia Artificial para identificar patrones de herencia y otros aspectos genéticos relevantes. Un nuevo aspecto por incluir en el árbol familiar es la definición de la identidad sexual (sexo y género), que cuenta con simbología definida^2^.

Dicha información genética es de gran utilidad e interés para la derivación de los pacientes y sus familias a los servicios de genética clínica de referencia que reivindicamos, de manera directa y sin intermediarios de la misma forma en la que se deriva a cirugía, cardiología y rehabilitación. También cabe destacar la necesidad de implantar más servicios de genética clínica de referencia a lo largo de toda la geografía española, pues existe un escaso número de los mismos. Esto ralentiza el diagnóstico y tratamiento de los pacientes, así como dificulta el abordaje de su riesgo de recurrencia.

Creemos firmemente que el médico de familia español tiene los suficientes conocimientos para poder derivar a un servicio de genética clínica^3^ si así lo precisaran sus pacientes, lo cual también reivindicamos y ponemos en valor con esta carta al director.

Todo esto parece ser consecuencia de que España sigue siendo el único país de la Unión Europea que carece de la especialidad sanitaria de Genética Clínica. Creemos firmemente que ha llegado el momento de que la creación de la especialidad sanitaria de Genética Clínica (para médicos, biólogos, farmacéuticos y químicos, con dos subprogramas: el clínico y el de laboratorio) sea una realidad. Sería el verdadero revulsivo para implantar toda esta serie de habilidades y competencias en la Atención Primaria española: la Medicina del Siglo XXI.

Los cuatro coautores de la presente carta al director declaran que no tienen responsabilidades éticas con respecto a la misma.

Ismael Ejarque Doménech.

Carolina Cuenca Valero.

Itziar Reinoso Fernández.

Ana María García Rodríguez.

## Financiación

Este trabajo no ha recibido ningún tipo de financiación.

## References

[1] Cruz-Cánovas M.C., Yanes-Rodríguez M., Gamero-de-Luna E.J. (2022). Genograma y árbol genealógico. Medicina de Familia. SEMERGEN..

[2] Bennett R.L., French K.S., Resta R.G., Austin J. (2022). Practice resource-focused revision: Standardized pedigree nomenclature update centered on sex and gender inclusivity: A practice resource of the National Society of Genetic Counselors. J Genet Couns..

[3] Ejarque Doménech I., Marín Reina P., García-Miñaur Rica S., Chirivella González I., Martínez Martínez M.T., García Rodríguez A.M. (2022). Criterios de derivación a genética clínica desde Atención Primaria. Documento de consenso. Aten Primaria..

